# Supplementary material for: Factors influencing the utilisation of anti-HBs titre testing services among nursing students in Northwest Ghana: A cross-sectional study
Source: PLOS Glob Public Health. 2024 Apr 25;4(4):e0003160. doi: 10.1371/journal.pgph.0003160 (PMC11045266; doi:10.1371/journal.pgph.0003160)
Supplement: Attachment — Submitted filename: Response to Reviewers.docx [file pgph.0003160.s002.docx]

**POINT-BY-POINT RESPONSE TO THE COMMENTS**

**Ref: Submission ID-** [PGPH-D-23-01827] - [EMID:7689901ae3037e3d]

**Title:** Factors influencing the utilisation of anti-HBs titre testing services among nursing students in Northwest Ghana: A cross-sectional study

**Journal Requirements:
Additional Editor Comments (if provided):**
Dear Authors:

We are pleased to inform you that we have finally received the reviewers' comments on your manuscript.

Also to the Reviewers' comments, you should consider these Editor's comments:

**General response:** I am very grateful for the insightful comments from the editor and the reviewers. I believe that your comments would help improve the quality of the manuscript, culminating in its publication.

**Specific editor’s comments**

1. Table 1: Discrepancies exist between the total population per class and the sum of individual rows. Please double-check all numerical values throughout the manuscript.

**Response:** I am very grateful for your critical comment. The total student population considered for sampling was 430, which was erroneously entered as 410. Therefore, 410 has been replaced with 430 (Please see Table 1 for the change effected). The entire manuscript has also been cross-checked. The error did not affect any part of the manuscript.

2. Tables 4 & 5: The analysis included fewer respondents than initially anticipated. Address the potential impact of this data loss on the precision of your results. Refer to these guidelines for more information: <https://www150.statcan.gc.ca/n1/en/catalogue/12-539-X> (pp. 1-49).

**Response:** I am very grateful for the insightful comment. The initial calculated sample size was 341. However, to account for non-responses and increase the representativeness of the study, the sample size was increase by 20% to 410. Also, the response rate in the study as reported in the results section was 98.2% and hence non-response rate of 1.80%. The 1.8% non-responders were contacted to ensure they did not differ from the responders in relation to the variables of interest in the study. Additionally, a deductive comparison of the population and sample, as evidenced by the data from Table 1 and Table 3, indicates no discrepancy in terms of representation. For example, in terms of representation, 56% of Wa NTC students represented both the population and respondents, 71.4% representation was also seen across board for first year students and 28.6% for second year students. Therefore, there is palpable evidence that no under-representation nor over-representation of the population occurred. As articulated by the Statistics Canada guidelines (2019) and Jensen et al. (2022), the potential for non-response bias is reduced drastically, since the data has a good coverage and representation, response rate, low non-response rate, and no identifiable differences between non-responders and responders. Based on these factors, I believe that the precision of the data was not affected. Below is the reference for Jensen et al. (2022);

Jensen HAR, Lau CJ, Davidsen M, Feveile HB, Christensen AI, Ekholm O. The impact of non-response weighting in health surveys for estimates on primary health care utilization. European Journal of Public Health. 2022;32: 450–455. doi:[10.1093/eurpub/ckac032](https://doi.org/10.1093/eurpub/ckac032)

3. Table 5: The combination of high OR values and wide confidence intervals suggests that logistic regression might not be the most suitable model for this analysis. The authors mention that logistic regression can overestimate results with low frequencies, as noted in <https://pubmed.ncbi.nlm.nih.gov/20213709/>. In such cases, it's recommended to consider Poisson or binomial regression models instead.

**Response:** Thank you for the insightful comment. Binary logistic regression modelling was used because the dependent variable of the study was binary categorical, with Yes or No as the two categories. To the best of my knowledge, Poisson regression models may be used when the dependent variable is a count data, which evidently does not fit into the data of this study. Furthermore, binomial regression (generalized linear) models may be used when the dependent variable is a continuous data with evidence of linearity. Based on the above, I think binary (binomial) logistic regression modeling was suitable for the analysis. Nevertheless, I have gained great insight from the suggestion of the academic editor. I have learned a new strategy for minimizing bias when handling sparse data or outcomes with rare occurrence as suggested by the recommended article (Heinze & Puhr, 2010). To significantly reduce bias and generate nearly bias-free estimates of odds ratios for sparse data or rare outcomes, I decided to run the firthlogit binary logistic regression analysis (Firth, 1993; Heinze & Puhr, 2010). Therefore, the large odds ratios and widened confidence intervals for hepatitis B vaccination and number of vaccine doses obtained previously have improved after running the firthlogit binary logistic regression model. Model fit indices (Penalized log likelihood, Akaike’s information criterion [AIC], Bayesian information criterion [BIC] and McFadden R^2^) were obtained using the firthfit function. Therefore, the analysis sub-section of the methods section has been updated. The hierarchical multivariate analyses have also been revised. Please see lines 193-200, 206-208 and Table 5. Once again, I am very grateful for this insight obtained from the suggestion of the academic editor.

**Reviewers' comments:**

**Reviewer #1:** While an effort has been made in the research, with a good sample size and laboratory tests, I consider there is no significant contribution to the topic. It is not specified which laboratory test and technique were used. It's unclear whether the titers of anti-HBsAg were determined or if the test was only qualitative. I believe the study's objective should be clarified, for example, whether there is a relationship between the titers of anti-HBsAg antibodies and the applied doses, age, gender, etc

**Response:** I am	very grateful for the comments. The objectives of the study cover, assessment of hepatitis B testing rate, hepatitis B vaccination rate, number of doses of hepatitis B vaccine received, anti-HBs titre testing rate and factors affecting the utilization of anti-HBs titre testing (Please see lines 82-85). This study is significant, as it is pioneering, being the first to explore the relationship between utilisation of anti-HBs titre testing and hepatitis B testing, hepatitis B vaccination, number of vaccine doses, and socio-demographics among nursing students in Ghana. With regards to the anti-HBs titres, the responses were self-reported and was assessed by asking respondent to answer with Yes or No, if they had done anti-HBs titre testing (Please see lines 167-168 of the manuscript). Indeed as part of determining the factors affecting the utilization of anti-HBs titre testing, various factors were considered namely socio-demographic characteristics (sex, age, marital status, religion, ethnicity, residential setting, name of college, programme of study, level of study and parental level of study), hepatitis B testing, Hepatitis B vaccination and number of vaccine doses received. However, after the chi square test bivariate level of analysis (Please see Table 3), sex, age, religion, ethnicity, residential setting, programme of study and level of study were not significant, and hence were not included in the multivariable analysis (Please see Table 5).

**Reviewer #2:** Your study, "Factors influencing the utilisation of anti-HBs titre testing services among nursing students in Northwest Ghana: A cross-sectional study," is well written and provides valuable insights into the hepatitis B vaccination and testing behaviors among nursing students in a specific region of Ghana.

**Response:** I am very grateful for your commendation on the current state of the manuscript. I hope to improve the quality of the manuscript with the constructive criticism and insights you have provided.

**Specific comments**

**Abstract**
1. The structured abstract provides a comprehensive and coherent overview of the study, from the background through to its conclusions. It effectively sets the stage for the full article by highlighting the significance of the research, its methodology, key findings, and implications. However, specifically after the introduction in the abstract, it would be beneficial to clearly articulate the objectives or aim of the study

**Response:** Thank you for this comment. The aim of the study was already captured in the abstract. However, as you critically observed, the aim was not stated explicitly. Therefore, based on your sound critique, I have revised the statement to read “The aim of the study was to determine the prevalence of hepatitis B testing, hepatitis B vaccination, number of vaccine doses received, socio-demographic characteristics, and their impact on anti-HBs titre testing among nursing students in Northwest Ghana.” (Please see lines 12-14 of the manuscript)

**Introduction**

2. While the introduction effectively outlines the significance of hepatitis B prevention and the need for research among nursing students in Northwest Ghana, it lacks explicit statements of research objectives.

**Response:** Thank you for the comment. The research objectives were indeed stated in the introduction section. However, like you rightly indicated, it lacked explicitness. I have therefore revised the statement on research objectives to read “Therefore, the objectives of this study were to assess the influence of hepatitis B testing, hepatitis B vaccination, the number of vaccine doses taken and relevant socio-demographics, on post-vaccination anti-HBs titre testing among nursing students in the resource-poor Northwest Ghana.”

**Methods**

3. For enhancing the geographical specificity and contextual understanding of your study's settings, it's recommended to include the latitude and longitude coordinates of the Wa and Lawra Nursing Training Colleges (NTCs).

**Response:** Thank you for the insightful comment. I have now included the geographical coordinates and boundaries of Upper West Region, Wa Municipality and Lawra Municipality. Please refer to lines 107-110 and lines 122 to 127 of the manuscript.

4. Clarifying the application of Cochran's formula for sample size calculation without accounting for the finite population of nursing students at Wa and Lawra NTCs is crucial, as adjustments or a correction factor may be needed to prevent overestimation. An explanation of whether a finite population correction was used and its impact on the sample size calculation would significantly enhance the methodological rigor.

**Response:** Thank you very much for this insightful comment. Cochran formula was applied on the basis that the total population of nursing students in the Upper West Region and Ghana at large was unknown, since such data are not readily available. Additionally, the total student population of Wa and Lawra NTCs were stated for purposes of stratification based on the probability proportional to stratal size strategy. However, based on your insightful comment, I have justified the basis for utilizing Cochran’s formula for determining the sample size of the study. The statement reads:

*Cochran’s formula was used since the total nursing student population in the Upper West Region and Ghana at large was unknown, as such data are not readily available. Please see lines 140-142.*

5. Additionally, justifying the decision to increase the sample size by 20% to account for non-responses would improve the study's methodological clarity. It's important to detail the basis for this adjustment, whether it stems from prior studies, pilot data, standard practices, or anticipated challenges specific to this research context.

**Response:** I am very grateful for the comment. The sample size was increased by 20% to account for non-responses, and based literature (Andrade, 2020) and the inherent low response rate associated with online surveys (Creswell & Creswell, 2018). Therefore, I have revised the statement to read;

*To account for non-responses, the inherent low response rate associated with e-surveys* (Creswell & Creswell, 2018) *and guided by Andrade* (2020)*, the sample size was increased by 20% to* ***410****.* Please see lines 146 & 147.

Ethical clearance

6. Could the author clarify whether, in addition to the granted institutional access by the Upper West Regional Health Director and the principals of the Wa and Lawra NTCs, there was a requirement or consideration for obtaining additional institutional ethical clearance, specifically from the institutions where the research was conducted?

**Response:** Thank you for the comment. As stated in the manuscript, ethical clearance (CHRPE/AP/392/20) was granted by the Committee on Human Research Publications and Ethics of the Kwame Nkrumah University of Science and Technology. Written institutional approvals from management of the two NTCs) as well as written informed consent were also obtained from the participants. Please see the ethical consideration subsection, specifically lines 213 to 218 of the manuscript.

**Discussion**

7. Lines 255 to 2587….Could the author consider comparing the high hepatitis B testing rate found among the nursing students in this study with similar populations of nursing students, if such data are available? This comparison could provide a more nuanced understanding of the health-seeking behaviors and awareness levels specific to nursing students, potentially highlighting unique factors influencing these rates in different educational or cultural settings

**Response:** Thank you for the comment. As suggested, the finding of high hepatitis B testing rate among the respondents have already been compared with other nursing student populations. This comparison reads:

*The study revealed a high hepatitis B testing rate among the nursing students. The high hepatitis B testing rate reflects proactive health-seeking behaviour and a demonstration of awareness of risks among the nursing students. Similar findings have been reported by Ma et al.* (2020) *among Vietnamese Americans. However, Osei et al.* (2019) *in Ghana and Kue and Thorburn* (2013) *in the USA reported lower hepatitis B testing rates. These differences in findings can be explained in terms of relative availability and access to testing services.* Please see lines 265-270.

8. Line 271 in what kind of population pls?

**Response:** Thank you for the comment. The populations have now been specified. The statement now reads:

*This finding corresponds with the reports of Paul and Peterside [36] in Nigeria, Ayalew and Horsa* (2017) *in Ethiopia, and Yuan et al.* (2019) *in China, who also reported low hepatitis B vaccination rates among medical students and healthcare workers.* Please see lines 279-280.

9. Lines 297 to 300: The finding that Wa NTC students in an urban area were significantly less likely to utilize post-vaccination anti-HBs titre testing services contradicts the typical assumption that urban settings, with their better access to healthcare facilities, would lead to higher service utilization. Could the author revisit this discussion to explore potential reasons or factors behind this unexpected pattern, without necessarily altering the study's findings?

**Response:** Thank you for the comment. Based on your insightful suggestion, the discussion which has been elaborated, now reads:

*However, students of Wa NTC were surprisingly statistically significantly less likely to utilise the post-vaccination anti-HBs titre testing services. Wa NTC is located in a predominantly urban locality and, therefore, is expected to be inequitably more advantaged in respect of availability and geographical accessibility to testing centres* (Levesque et al., 2013)*. However, considering that post-vaccination anti-HBs titre testing is generally unavailable in the UWR, these factors probably become generally less important explainable factors. Additionally, rural localities are relatively advantaged in terms of outreach services by non-profit organizations because of their pro-poor nature. Therefore, the rural based Lawra NTC may have benefited from financial assistance from these organizations towards utilization of post-vaccination anti-HBs titer testing.*

*However, further research is required to explore other factors that favour rural localities with respect to post-vaccination anti-HBs titre testing.* Please see lines 307-317

10. Line 307 pls specify the pop.

**Response:** I am grateful for the comment. The population has now been specified. The statement now reads:

*This finding is in similitude with the findings of Mora and Trapero* (2018) *in Spain among children enrolled in a vaccination programme.* Please see line 319-320.

11. Lines 331 to 333 the first in Ghana or globally?

**Response:** Thank you for the comment. The population has now been specified. The statement now reads:

*This study is pioneering, being the first to explore the relationship between utilisation of anti-HBs titre testing and hepatitis B testing, hepatitis B vaccination, number of vaccine doses, and socio-demographics among nursing students in Ghana.* Please see lines 344-346
